# Population attributable fractions of cardiovascular diseases linked to lifestyle behaviors: the ATTICA study (2002–2022)

**DOI:** 10.3389/fcvm.2025.1551020

**Published:** 2025-10-01

**Authors:** Evangelia Damigou, Costas Anastasiou, Konstantina Kyrili, Fotios Barkas, Evangelos Liberopoulos, Evangelinos Michelis, Christos Pitsavos, Petros P. Sfikakis, Costas Tsioufis, Demosthenes Panagiotakos

**Affiliations:** ^1^ Department of Nutrition and Dietetics, School of Health Sciences and Education, Harokopio University, Athens, Greece; ^2^ Department of Internal Medicine, Medical School, University of Ioannina, Ioannina, Greece; ^3^ First Department of Propaedeutic Internal Medicine, Medical School, National and Kapodistrian University of Athens, Laiko General Hospital, Athens, Greece; ^4^ First Cardiology Clinic, Medical School, National and Kapodistrian University of Athens, Athens, Greece

**Keywords:** cardiovascular disease, population attributable fraction, generalized impact fraction, lifestyles, modifiable factors

## Abstract

**Introduction:**

The stagnant cost of cardiovascular disease (CVD) can be diminished with the effective management of well-known lifestyle factor modifications. However, when it comes to treating the individual and not the disease, research on these factors and their interactions is limited.

**Aim:**

The purpose of this study was to evaluate the number of CVD cases that would be prevented in males/females and younger/older participants if specific lifestyle patterns were managed.

**Methods:**

The sample was 1,988 (mean age: 45 ± 14 years, 49.7% male) individuals from the ATTICA cohort study (2002–2022), who were initially free-of-CVD. Trained health professionals evaluated combined fatal/non-fatal CVD outcomes, 2 major non-modifiable risk factors (i.e., sex and age) and 6 categories of modifiable risk factors [i.e., low/middle socio-economic status (SES), urban residence, at least one clinical, one psychological or one unhealthy lifestyle factor]. Population attributable fractions (PAF) and generalized impact fractions (GIFs) (percentage of exposure removal: 70%), were computed for each sole factor, as well as different combinations of these factors, representing different lifestyle patterns.

**Results:**

A lifestyle pattern comprising of having a low/middle SES, urban residence, at least one clinical, psychological and unhealthy lifestyle factor was associated with a PAF of 82%; 8 out of 10 CVD cases would have been prevented if all these factors had been completely managed in the sample. The respective PAF and GIF was similar in males and females but differed significantly based on the age of the participants. PAF varied between 69% and 80%, when participants had all 5 factor categories, but were healthy (with no clinical factors) or of high SES, respectively.

**Conclusion:**

The increased burden of CVD could be significantly reduced with tailored programs focusing on managing lifestyle patterns, especially in individuals (males or females) older than 45 years-old.

## 1 Introduction

Based on the most up-to-date evidence, in 2021, cardiovascular disease (CVD) cost the European Union (EU) an estimated €282 billion annually, representing 2% of Gross Domestic Product (GDP) (1), highlighting the stagnant cost that CVD pose on population health. In epidemiological research, population attributable fraction (PAF) is commonly used to quantify disease burden, which is affected by the strength of the risk factor (usually represented by relative risk), as well as the prevalence of that factor in the population (2). Differences in risk factor prevalence and/or strength exist in the population, based on non-modifiable characteristics such as age, sex and race (3).

Robust research has identified specific modifiable CVD risk factors, such as diabetes, hypertension, hypercholesterolemia, excess weight, smoking, physical inactivity, unhealthy dietary habits and the CVD burden accompanying these factors (4, 5). Furthermore, CVD risk factors interact with each other, with synergistic or antagonistic effects, therefore, it might be prudent to focus on lifestyle patterns and not only specific risk factors (3). It is well-known that a healthy lifestyle, associated with lower CVD risk and all-cause mortality, encompasses taking medications to manage clinical factors, abstaining from smoking, eating healthy, being physically active, and taking care of our physical and mental health (4–9). This might include having -physical and economical- access to healthcare facilities and professionals, which by extent, might be more easily achieved in urban areas of higher socio-economic status (SES) (10). However, usually, all these behaviors cannot be adopted concomitantly; more often than not, even single lifestyle changes might be proven difficult to follow (11). Quantifying the CVD burden that could be prevented by modifying different risk factors in the context of different lifestyles (i.e., altering a risk factor while taking into account the rest of an individual's behaviors), is particularly important for effective goal setting (3, 7).

Data on lifestyle patterns (with different combinations of beneficial and detrimental factors) and their burden associated with CVD are still inadequate. Therefore, the aim of this study was to evaluate different lifestyle patterns to pinpoint which factor(s) should be prioritized for effective and tailored interventions. In order to rigorously analyze most well-known CVD risk factors, but retain the ability to generalize results, we categorized risk factors as: (i) low/middle SES, (ii) urban residence, (iii) having an increased body mass index (BMI), (iv) at least one clinical CVD risk factor (i.e., diabetes, hypercholesterolemia, hypertension, chronic kidney disease-CKD), (v) at least one psychological factor (i.e., anxiety, depression), (vi) at least one unhealthy lifestyle factor (i.e., smoking, physical inactivity, low diet quality).

## 2 Materials and methods

### 2.1 Study design

The ATTICA study is a prospective epidemiological cohort study that gathered population-based data. Its primary objectives were to determine the prevalence and incidence of CVD and to examine various socio-demographic, biochemical, clinical, anthropometric, lifestyle, and psychological factors associated with CVD occurrence. Comprehensive details on the study's objectives, methods and procedures can be found in previously published papers (12–14).

### 2.2 Bioethics

The ATTICA study complied with the principles outlined in the Declaration of Helsinki (1989) by the World Medical Association. It received approval from the Institutional Ethics Committee of Athens Medical School (#017/1.5.2001) and the Bioethics Committee of Harokopio University (#38/29.03.2022). All participants were fully informed about the study's objectives and procedures and provided written consent before participation.

### 2.3 Study setting

The study took place in the Attica region, which consists of 78% urban areas, including Athens, the capital of Greece. Participants were randomly chosen using city polls. To achieve representativeness, the sampling was stratified by age and sex in line with the 2001 census data for each city.

### 2.4 Study participants and sample size

**Figure 1:**
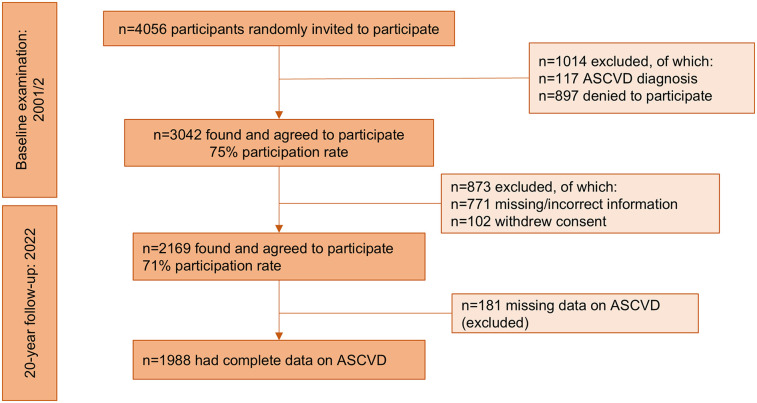
Diagram depicting the flow of participants in the ATTICA study (*n* = 1,988).

Of the 4,056 individuals initially invited, 3,042 met the study criteria and willingly participated in the study (75% participation rate). Individuals who took part in the study were not residing in institutions. Participants with a prior history of CVD, cancer, or other inflammatory conditions were excluded. Detailed inclusion and exclusion criteria are outlined in the baseline paper (12). By 2022, 1,988 (mean age: 45 ± 14 years, 49.7% male) of the original 3,042 participants were found, had complete data for CVD assessment, and were, thus, the working sample for this study (Figure 1). No significant differences in the age and sex distribution between the current sample and the baseline sample were observed (all *p*-values > 0.05).

### 2.5 Study variables and measurements

#### 2.5.1 Exposures/risk factors

##### 2.5.1.1 Sociodemographic factors

At baseline, age and sex were recorded. Participants were stratified into two groups, i.e., those younger than 45 years and those 45 years or older, based on the median baseline age of the study sample. Area of residence was categorized as urban vs. rural (i.e., an urban area is defined as any municipality or commune whose most populous settlement exceeds 2,000 inhabitants, while those whose most populous settlement has fewer than 2,000 residents are classified as rural) and socio-economic status (SES) was categorized (based on mean annual income and education level) as low, middle and high, based on previously described methodology (15).

##### 2.5.1.2 Clinical factors

Clinical factors were assessed by trained health professionals (12–14). Hypertension was defined as an average systolic/diastolic blood pressure (measured by the ELKA aneroid manometric sphygmometer, Von Schlieben Co, West Germany) >140/90 mmHg or use of antihypertensive drugs. Hypercholesterolemia was characterized by total cholesterol levels of ≥200 mg/dl and/or the use of cholesterol-lowering drugs. Type 2 diabetes mellitus was defined as a fasting plasma glucose level of ≥126 mg/dl or the use of antidiabetic medications. CKD was defined as an estimated glomerular filtration rate (eGFR) (based on the Chronic Kidney Disease Epidemiology Collaboration equation) <60 ml/min/1.73 m^2^, signifying mild to moderate kidney damage (16).

##### 2.5.1.3 Anthropometric factors

Weight was recorded to the nearest 0.1 kg, and height to the nearest 0.5 cm, using standard measurement procedures. BMI was calculated by dividing weight by the square of height. The cumulative average BMI of all measurements during 2002–2022 was computed. Increased BMI was defined as a BMI exceeding 25 kg/m² (i.e., including overweight and obesity).

##### 2.5.1.4 Lifestyle factors

Dietary habits were evaluated using a validated 156-item semi-quantitative food frequency questionnaire (FFQ) validated for the Greek population (17). Adherence to the Mediterranean diet was assessed using the MedDietScore, an evidence-based score (range: 0–55), which includes 11 items; 7 traditional Mediterranean foods (fruits, vegetables, whole grains, potatoes, legumes, fish, and olive oil), scored positively (0–5, from very rare to very frequent consumption), 3 non-traditional Mediterranean food groups (full-fat dairy, poultry, and red meat), scored inversely, and alcohol which is evaluated on a non-linear scale (0 for no or >7 servings/day; 1–5 for intermediate consumption levels) (18). The mean MedDietScore during the baseline and follow-up examinations, 2002–2022, was calculated and, based on the median value of 25, participants were categorized as having low, or high adherence to the Mediterranean diet. Participants were classified according to their smoking status as (current) smokers or non-current smokers. Physical activity was assessed using the validated short-form International Physical Activity Questionnaire (IPAQ) (19); based on which participants were classified as physically inactive or active.

#### 2.5.2 Outcome: cardiovascular disease assessments

CVD events were assessed at all follow-up examinations (2006, 2012, and 2022) by trained personnel, using the International Classification of Diseases (ICD)-10 criteria. The ICD codes applied included: WHO-ICD codes 410–414.9, 427.2, and 427.6 for acute myocardial infarction, unstable angina, and other forms of ischemia; WHO-ICD codes 400.0–404.9, 427.0–427.5, and 427.9 for various types of heart failure and chronic arrhythmias; and WHO-ICD codes 430–438 for stroke.

### 2.6 Statistical analysis

Categorical variables are presented as relative frequencies and associations between these variables were taken from the chi-squared test. Quantitative variables are presented as mean and standard deviation; differences between mean age and CVD status over the 20-year period were analyzed using Student's *t*-test. PAF, referring to the percentage of CVD cases that would have been prevented if a certain risk factor had been completely eradicated from the sample, was computed via the Miettinen formula (1974) (20):

$$PAF=P_{c}\;\frac{RR-1}{RR}$$

where P_c_: is the prevalence of exposure among cases, RR: relative risk of developing a CVD event during 2002–2022. Moreover, Generalized Impact Fractions (GIF), referring to the percentage of CVD cases that would have been prevented if a risk factor had been reduced to a certain percentage, but not completely eradicated from the sample, was computed *via* the formula (2):

GIF=Pr*PAF

where Pr: percentage of exposure removal (i.e., 70% for all studied factors). Studied risk factors included low/middle SES, urban residence, increased BMI, the presence of at least one clinical factor, at least one unhealthy lifestyle factor, and at least one psychological factor. Relative risks for the PAF formula were taken as approximates of hazard ratios estimated from Cox proportional hazards models, with CVD events recorded annually. All models were adjusted for age, sex, SES, area of residence, clinical factors, i.e., hypertension, hypercholesterolemia, diabetes mellitus, CKD (which partially account for the impact of antihypertensive and lipid-lowering medications), BMI, lifestyle factors (i.e., smoking, physical activity status, Mediterranean diet adherence). Proportionality was graphically assessed. Stratified analyses by sex and age were performed on the basis of significant interactions. For the statistical analyses, STATA version 17 (STATA Corp, College Station, Texas, USA) was used.

## 3 Results

As it has already been reported in previous publications, over the 20-year follow-up period, 718 participants (36%) experienced a combined fatal or non-fatal CVD event (40% in males, 32% in females, *p* for gender difference <0.001). Among these events, 71.7% were coronary heart disease, 4.3% were strokes, and 24% were classified as other CVDs (14).

### 3.1 Participant characteristics by CVD status

Participant characteristics by CVD status in 2022 are presented in Table 1. Briefly, participants who developed a CVD event during the 20-year follow-up were older, males, of low or middle SES, urban residents, affected by at least one clinical factor (i.e., hypertension, hypercholesterolemia, diabetes or CKD), had an increased BMI, and had adopted at least one unhealthy lifestyle factor (i.e., smoking, physical inactivity, low diet quality). In Table 1, these characteristics are also presented by sex and age group.

**Table 1:** Descriptive characteristics of the ATTICA study sample by cardiovascular disease status in 2022, in the total sample, as well as in males/females and younger/older participants (*n* = 1,988).

Characteristics	CVD status at 2022		*p*-value*
*Total sample*	*CVD-free in 2022*	*CVD in 2022*	
Age ± SD, years	38 ± 9.0	58 ± 11	<0.001
Males, %	46	55	<0.001
Low/Middle SES, %	61	75	<0.001
Urban residence, %	75	83	<0.001
At least one clinical factor, %	47	88	<0.001
Increased BMI, %	57	74	<0.001
At least one unhealthy lifestyle factor, %	87	94	<0.001
At least one psychological factor, %	51	60	0.081
*Males*			
Age ± SD, years	38 ± 8.3	57 ± 11	<0.001
Low/Middle SES, %	56	69	0.001
Urban residence, %	73	82	0.001
At least one clinical factor, %	54	87	<0.001
Increased BMI, %	72	79	0.127
At least one unhealthy lifestyle factor, %	92	96	0.022
At least one psychological factor, %	48	58	0.156
*Females*			
Age ± SD, years	37 ± 9.5	59 ± 10	<0.001
Low/Middle SES, %	64	83	<0.001
Urban residence, %	75	83	0.007
At least one clinical factor, %	35	90	<0.001
Increased BMI, %	44	67	<0.001
At least one unhealthy lifestyle factor, %	82	90	0.004
At least one psychological factor, %	53	65	0.188
*Age <45 years*			
Age ± SD, years	35 ± 7.6	37 ± 5.9	0.004
Males, %	46	68	0.001
Low/Middle SES, %	59	63	0.618
Urban residence, %	73	82	0.132
At least one clinical factor, %	43	71	0.002
Increased BMI, %	54	73	0.009
At least one unhealthy lifestyle factor, %	86	98	0.004
At least one psychological factor, %	51	56	0.691
*Age* ≥*45 years*			
Age ± SD, years	49 ± 2.6	60 ± 9.0	<0.0001
Males, %	48	54	0.096
Low/Middle SES, %	67	76	0.029
Urban residence, %	79	83	0.186
At least one clinical factor, %	66	90	<0.001
Increased BMI, %	72	75	0.549
At least one unhealthy lifestyle factor, %	91	93	0.361
At least one psychological factor, %	48	61	0.128
*Males <45 years*			
Age ± SD, years	35 ± 7.2	38 ± 6.0	0.033
Low/Middle SES, %	55	62	0.467
Urban residence, %	73	82	0.166
At least one clinical factor, %	51	72	0.028
Increased BMI, %	71	79	0.298
At least one unhealthy lifestyle factor, %	92	100	0.058
At least one psychological factor, %	49	54	0.738
*Males* ≥*45 years*			
Age ± SD, years	48 ± 1.5	59 ± 9.2	<0.001
Low/Middle SES, %	62	70	0.156
Urban residence, %	76	82	0.158
At least one clinical factor, %	67	89	<0.001
Increased BMI, %	75	79	0.533
At least one unhealthy lifestyle factor, %	93	96	0.347
At least one psychological factor, %	42	59	0.133
*Females <45 years*			
Age ± SD, years	34 ± 7.9	37 ± 5.8	0.110
Low/Middle SES, %	63	67	0.805
Urban residence, %	74	81	0.476
At least one clinical factor, %	30	60	0.146
Increased BMI, %	40	59	0.111
At least one unhealthy lifestyle factor, %	80	95	0.086
At least one psychological factor, %	53	67	0.636
*Females* ≥*45 years*			
Age ± SD, years	50 ± 3.1	60 ± 8.7	<0.001
Low/Middle SES, %	72	84	0.029
Urban residence, %	81	83	0.610
At least one clinical factor, %	64	92	<0.001
Increased BMI, %	67	69	0.794
At least one unhealthy lifestyle factor, %	89	89	0.839
At least one psychological factor, %	55	65	0.441

BMI, body mass index; CVD, cardiovascular disease; SD, standard deviation; SES, socio-economic status.

* *P*-values refer to differences between participants with and without a CVD event at 2022 and were taken from chi-squared tests for all variables, with the exception of age (a *t*-test was used in this case).

### 3.2 Population attributable fractions of CVD in relation to modifiable risk factors

Table 2 shows PAFs and GIFs of CVD cases in relation to modifiable risk factors by sex and age in the ATTICA study sample. Of note, approximately 4 out of 10 CVD cases (95% CI: 33%, 46%) would have been prevented if participants were healthy, without clinical factors. Alternatively, 3 out of 10 CVD cases (23%, 32%) would have been prevented if clinical factors had been reduced by 70%. Moreover, 3 out of 10, or 2 out of 10 CVD cases, would have been prevented if increased BMI had been completely or partly (at a 70% percentage of exposure removal) managed in the population, respectively (Table 2). And, similarly, 3 out of 10, or 2 out of 10 CVD cases, would have been prevented if unhealthy lifestyles had been completely or partly managed in the population, respectively (Table 2). Variations in the PAFs and GIFs by sex and age can be seen in Table 2.

**Table 2:** Population attributable fractions (PAF) and generalized impact fractions (GIF) of cardiovascular diseases in relation to different modifiable risk factors by sex and age in the ATTICA study (*n* = 1,988).

Modifiable risk factors	PAF, % (95% CI)*	GIF, % (95% CI)**
*Total sample*		
Low/Middle SES	9.2 (2.4, 14)	6.4 (1.7, 9.7)
Urban residence	17 (0.63, 30)	12 (0.44, 21)
At least one clinical factor	41 (33, 46)	29 (23, 32)
Increased BMI	30 (8.8, 38)	21 (6.1, 27)
At least one unhealthy lifestyle factor	33 (2.4, 52)	23 (1.7, 36)
At least one psychological factor	12 (0, 21)	8.5 (0, 14)
*Males*		
Low/Middle SES	13 (0, 25)	9.4 (0, 17)
Urban residence	21 (0, 35)	15 (0, 25)
At least one clinical factor	40 (28, 47)	28 (20, 33)
Increased BMI	16 (0, 39)	11 (0, 27)
At least one unhealthy lifestyle factor	1.9 (0.05, 3.2)	1.4 (0.04, 2.2)
At least one psychological factor	12 (0, 21)	8.1 (0, 14)
*Females*		
Low/Middle SES	28 (0.58, 42)	20 (0.40, 29)
Urban residence	10 (0, 31)	7.6 (0, 22)
At least one clinical factor	40 (27, 45)	28 (19, 31)
Increased BMI	21 (0, 30)	15 (0, 21)
At least one unhealthy lifestyle factor	1.8 (0, 10)	1.3 (0, 7.3)
At least one psychological factor	12 (0, 26)	8.4 (0, 18)
*Age <45 years*		
Low/Middle SES	8.6 (0, 23)	6.0 (0, 16)
Urban residence	24 (0, 41)	16 (0, 28)
At least one clinical factor	20 (6.1, 26)	14 (4.2, 18)
Increased BMI	16 (0, 31)	11 (0, 22)
At least one unhealthy lifestyle factor	6.1 (0.75, 9.2)	4.3 (0.52, 6.4)
At least one psychological factor	6.7 (0, 21)	4.7 (0, 15)
*Age* ≥*45 years*		
Low/Middle SES	24 (5.7, 36)	17 (4.1, 25)
Urban residence	15 (0, 31)	10 (0, 21)
At least one clinical factor	59 (47, 66)	41 (33, 46)
Increased BMI	42 (0, 52)	29 (0, 36)
At least one unhealthy lifestyle factor	3.5 (0, 5.4)	2.4 (0, 3.8)
At least one psychological factor	14 (0, 24)	10 (0, 17)
*Males <45 years*		
Low/Middle SES	8.9 (0, 23)	6.2 (0, 16)
Urban residence	25 (0, 43)	17 (0, 30)
At least one clinical factor	23 (2.8, 31)	16 (2.0, 29)
Increased BMI	21 (0, 41)	14 (0, 29)
At least one unhealthy lifestyle factor	41 (14, 59)	29 (10, 41)
At least one psychological factor	4.8 (0, 20)	3.3 (0, 14)
*Males* ≥*45 years*		
Low/Middle SES	17 (0, 31)	11 (0, 21)
Urban residence	20 (0, 28)	14 (0, 26)
At least one clinical factor	56 (38, 65)	39 (27, 45)
Increased BMI	13 (0, 38)	8.8 (0, 27)
At least one unhealthy lifestyle factor	5.9 (0, 25)	4.1 (0, 17)
At least one psychological factor	15 (0, 244)	10 (0, 17)
*Females <45 years*		
Low/Middle SES	6.6 (0, 32)	4.6 (0, 23)
Urban residence	20 (0, 47)	14 (0, 32)
At least one clinical factor	13 (0, 18)	9.4 (0, 13)
Increased BMI	13 (0, 20)	9.0 (0, 14)
At least one unhealthy lifestyle factor	11 (0, 43)	7.8 (0, 30)
At least one psychological factor	15 (0, 32)	11 (0, 23)
*Females* ≥*45 years*		
Low/Middle SES	36 (4.5, 53)	26 (3.1, 37)
Urban residence	8.6 (0, 34)	6.0 (0, 24)
At least one clinical factor	65 (43, 72)	46 (30, 51)
Increased BMI	3.8 (0, 26)	2.7 (0, 18)
At least one unhealthy lifestyle factor	3.2 (0, 17)	2.2 (0, 12)
At least one psychological factor	13 (0, 30)	9.0 (0, 21)

BMI, body mass index; CI, confidence interval; CVD, cardiovascular disease; GIF, generalized impact fraction; PAF, population attributable fraction; RR, relative risk; SES, socio-economic status.

* PAF refers to the percentage of CVD cases that would have been prevented if each risk factor (presented in each line) had been completely eradicated from the sample. It was computed via the Miettinen formula (1974): $PAF=P_{c}\;\frac{RR-1}{RR}$, where Pc: is the prevalence of exposure among cases, RR: relative risk of developing a CVD event during 2002–2022, taken from Cox proportional hazards models, adjusted for age, sex, SES, area of residence, clinical factors (i.e., hypertension, hypercholesterolemia, diabetes mellitus, chronic kidney disease), BMI, lifestyle factors (smoking, physical activity status, Mediterranean diet adherence).

** GIF refers to the percentage of CVD cases that would have been prevented if each risk factor (presented in each line) had been reduced to a certain percentage, but not completely eradicated from the sample. It was computed via the formula: GIF=Pr*PAF, where Pr: percentage of exposure removal (i.e., in this case, 70% for all factors).

### 3.3 PAFs attributed to different lifestyle patterns

Combined PAFs of CVD cases in relation to combinations of modifiable risk factors, representing lifestyle patterns, by sex and age, are depicted in Table 3. A lifestyle pattern comprising of having a low/middle SES, urban residence, at least one clinical, psychological and unhealthy lifestyle factor was associated with a PAF of 82%; 8 out of 10 CVD cases would have been prevented if all these factors had been completely managed in the sample. The respective PAF was similar in males and females but differed significantly based on the age of the participants (Table 3). For lifestyle patterns comprising of all 6 aforementioned factor categories, except for one, PAF varied between 69% and 80%, when participants had all 5 factors, but were healthy (with no clinical factors) or of high SES, respectively. More modifiable risk factor combinations and their variations in PAFs, by sex and age, can been seen in detail in Table 3.

**Table 3:** Combined population attributable fractions (PAF) of cardiovascular diseases in relation to combinations of modifiable risk factors, representing lifestyle patterns, in the ATTICA study by sex and age (*n* = 1,988).

	Modifiable risk factors						Combined PAF*								
Lifestyle patterns	Low, Middle SES	Urban residence	Clinical factor (≥1)	Increased BMI	Unhealthy lifestyle factor (≥1)	Psycho-logical factor (≥1)	Total sample	Males	Females	Age < 45years	Age ≥ 45years	Males <45 years	Males ≥45 years	Females <45years	Females ≥45 years
Low/Middle SES + urban	yes	yes	no	no	no	no	25%	31%	35%	31%	35%	32%	34%	25%	42%
Low/Middle SES + urba*n* + clinical factor (≥1)	yes	yes	yes	no	no	no	56%	59%	61%	44%	74%	47	71%	35%	80%
Low/Middle SES + urba*n* + clinical factor (≥1) + increased BMI	yes	yes	yes	yes	no	no	69%	65%	69%	53%	85%	58%	75%	43%	80%
Low/Middle SES + urban + clinical factor (≥1) + increased BMI + unhealthy factor (≥1)	yes	yes	yes	yes	yes	no	79%	66%	70%	56%	85%	75%	76%	49%	81%
All factors	yes	yes	yes	yes	yes	yes	82%	70%	73%	58%	87%	76%	79%	57%	83%
All factors but high SES	no	yes	yes	yes	yes	yes	80%	66%	63%	54%	83%	74%	75%	54%	74%
All factors but rural	yes	no	yes	yes	yes	yes	78%	62%	71%	45%	85%	68%	74%	46%	81%
All factors but no clinical factors	yes	yes	no	yes	yes	yes	69%	50%	56%	48%	69%	69%	53%	50%	52%
All factors but healthy weight	yes	yes	yes	no	yes	yes	74%	64%	66%	50%	78%	70%	76%	50%	82%
All factors but healthy lifestyle	yes	yes	yes	yes	no	yes	73%	69%	73%	55%	87%	60%	78%	51%	82%
All factors but high SES and no psychological factors	no	yes	yes	yes	yes	no	77%	61%	58%	52%	80%	73%	71%	46%	70%
All factors but rural and high SES	no	no	yes	yes	yes	yes	76%	56%	59%	40%	80%	65%	69%	42%	71%
Increased BMI, Unhealthy lifestyle factor (≥1), psychological factor (≥1), but no other factors	no	no	no	yes	yes	yes	59%	27%	31%	25%	52%	55%	30%	34%	18%
Unhealthy lifestyle factor (≥1), and psychological factor (≥1), but no other factors	no	no	no	no	yes	yes	41%	14%	14%	10%	17%	43%	20%	24%	15%
Urban + clinical factor (≥1), but no other factors	no	yes	yes	no	no	no	51%	53%	46%	39%	65%	42%	64%	30%	68%
Clinical factor (≥1), increased BMI, but no other factors	no	no	yes	yes	no	no	59%	50%	53%	33%	76%	39%	61%	24%	66%
Increased BMI, unhealthy lifestyle factor (≥1), but no other factors	no	no	no	yes	yes	no	53%	18%	22%	21%	44%	53%	18%	22%	6.8%

BMI, body mass index; CI, confidence interval; CVD, cardiovascular disease; PAF, population attributable fraction; RR, relative risk; SES, socio-economic status.

* PAF refers to the percentage of CVD cases that would have been prevented if each risk factor (presented in each line) had been completely eradicated from the sample. It was computed via the Miettinen formula (1974): $PAF=P_{c}\;\frac{RR-1}{RR}$, where Pc: is the prevalence of exposure among cases, RR: relative risk of developing a CVD event during 2002–2022, taken from Cox proportional hazards models, adjusted for age, sex, SES, area of residence, clinical factors (i.e., hypertension, hypercholesterolemia, diabetes mellitus, chronic kidney disease), BMI, lifestyle factors (smoking, physical activity status, Mediterranean diet adherence). For the computation of the combined PAFs, the formula used was: $Combined\;PAF=\;\;1-\;\overset{n}{\underset{i=1}{\prod }}\;(1-PAFi)$.

### 3.4 GIFs attributed to different lifestyle patterns

Combined GIFs of CVD cases in relation to combinations of modifiable risk factors, representing lifestyle patterns, by sex and age, are depicted in Table 4. Results are similar to Table 3 but concern a more realistic scenario of partly managing SES, area of residence, BMI, clinical, lifestyle and psychological factors, at a percentage of exposure removal of 70%.

**Table 4:** Combined generalized impact fractions (GIF) of cardiovascular diseases in relation to combinations of modifiable risk factors, representing lifestyle patterns, in the ATTICA study by sex and age (*n* = 1,988).

	Modifiable risk factors						Combined GIF*								
Lifestyle patterns	Low, Middle SES	Urban residence	Clinical factor (≥1)	Increased BMI	Unhealthy lifestyle factor (≥1)	Psycho-logical factor (≥1)	Total sample	Males	Females	Age < 45years	Age ≥ 45years	Males <45 years	Males ≥45 years	Females <45years	Females ≥45 years
Low/Middle SES + urban	yes	yes	no	no	no	no	18%	23%	26%	21%	325%	22%	23%	17%	30%
Low/Middle SES + urban + clinical factor (≥1)	yes	yes	yes	no	no	no	42%	45%	47%	32%	56%	34%	53%	25%	62%
Low/Middle SES + urban + clinical factor (≥1) + increased BMI	yes	yes	yes	yes	no	no	54%	51%	55%	40%	69%	43%	57%	32%	63%
Low/Middle SES + urban + clinical factor (≥1) + increased BMI + unhealthy factor (≥1)	yes	yes	yes	yes	yes	no	64%	51%	55%	42%	69%	60%	59%	37%	64%
All factors	yes	yes	yes	yes	yes	yes	67%	55%	59%	45%	73%	61%	63%	44%	67%
All factors but high SES	no	yes	yes	yes	yes	yes	65%	51%	49%	41%	67%	58%	58%	41%	56%
All factors but rural	yes	no	yes	yes	yes	yes	63%	47%	56%	34%	69%	53%	57%	35%	65%
All factors but no clinical factors	yes	yes	no	yes	yes	yes	54%	38%	43%	36%	53%	54%	39%	38%	39%
All factors but healthy weight	yes	yes	yes	no	yes	yes	59%	50%	52%	38%	61%	55%	59%	39%	66%
All factors but healthy lifestyle	yes	yes	yes	yes	no	yes	58%	55%	59%	42%	72%	45%	61%	39%	66%
All factors but high SES and no psychological factors	no	yes	yes	yes	yes	no	65%	51%	49%	41%	67%	58%	58%	41%	56%
All factors but rural and high SES	no	no	yes	yes	yes	yes	60%	42%	45%	30%	63%	50%	51%	32%	53%
Increased BMI, Unhealthy lifestyle factor (≥1), psychological factor (≥1), but no other factors	no	no	no	yes	yes	yes	44%	19%	23%	19%	38%	40%	21%	25%	13%
Unhealthy lifestyle factor (≥1), and psychological factor (≥1), but no other factors	no	no	no	no	yes	yes	30%	9.4%	9.6%	8.8%	12%	31%	13%	17%	11%
Urban + clinical factor (≥1), but no other factors	no	yes	yes	no	no	no	38%	39%	33%	28%	47%	30%	47%	22%	49%
Clinical factor (≥1), increased BMI, but no other factors	no	no	yes	yes	no	no	44%	36%	39%	23%	58%	27%	44%	17%	47%
Increased BMI, unhealthy lifestyle factor (≥1), but no other factors	no	no	no	yes	yes	no	39%	12%	16%	15%	31%	38%	12%	16%	4.8%

BMI, body mass index; CI, confidence interval; CVD, cardiovascular disease; GIF, generalized impact fraction; PAF, population attributable fraction; RR, relative risk; SES, socio-economic status.

* GIF refers to the percentage of CVD cases that would have been prevented if each risk factor (presented in each line) had been reduced to a certain percentage, but not completely eradicated from the sample. It was computed via the formula: GIF=Pr*PAF, where Pr: percentage of exposure removal (i.e., in this case, 70% for all factors), PAF: the percentage of CVD cases that would have been prevented if each risk factor (presented in each line) had been completely eradicated from the sample. It was computed via the Miettinen formula (1974): $PAF=P_{c}\;\frac{RR-1}{RR}$, where Pc: is the prevalence of exposure among cases, RR: relative risk of developing a CVD event during 2002–2022, taken from Cox proportional hazards models, adjusted for age, sex, SES, area of residence, clinical factors (i.e., hypertension, hypercholesterolemia, diabetes mellitus, chronic kidney disease), BMI, lifestyle factors (smoking, physical activity status, Mediterranean diet adherence).

For the computation of the combined GIFs, the formula used was: $Combined\;GIF=\;\;1-\;\overset{n}{\underset{i=1}{\prod }}\;(1-GIFi)$.

Approximately 7 out of 10 CVD cases for the total sample, 5 for males, 6 for females, 5 for participants younger than 45 years-old, and 7 for participants older than 45 years-old, would have been prevented if all factors had been managed at a percentage of 70% (Table 4).

## 4 Discussion

### 4.1 Main findings

This study assessed the potential reduction in CVD cases that could have been achieved if specific modifiable factors—including low or middle socioeconomic status (SES), urban residence, elevated body mass index (BMI), and the presence of at least one clinical, psychological, or unhealthy lifestyle factor—had been effectively addressed, either fully or partially, within the population. Additionally, the analysis considered the combined effects of these factors, representing various lifestyle patterns, to estimate their collective impact. The evaluation was conducted for the overall population, as well as stratified by sex (males and females) and age groups (younger and older participants), to provide insights into demographic-specific outcomes. The findings revealed that a significant proportion of CVD cases could potentially be prevented through effective management of the studied modifiable factors. Specifically, if these factors were fully addressed and eliminated from the population, approximately 8 out of every 10 CVD cases could have been avoided. Even with a partial reduction, corresponding to the removal of 70% of exposure to these factors, about 7 out of every 10 cases could still have been prevented. These results highlight the substantial impact that targeted interventions and public health strategies aimed at addressing modifiable risk factors could have on reducing the burden of CVD in the population.

Targeting the CVD burden is crucial due to its significant impact on public health, healthcare systems, and economic stability (1, 4). Addressing the CVD burden aligns with global initiatives, such as the Sustainable Development Goals (SDGs), which aim to reduce premature mortality from non-communicable diseases (NCDs) by promoting health and well-being (21). Moreover, with increasing life expectancy, the global population is aging, leading to a higher prevalence of CVD (22). Proactive measures are essential to manage this growing burden. Our results on combined PAFs and GIFs associated with lifestyle patterns, propose that, in public health actions, programs to reduce the burden of CVD might need to be tailored to the lifestyle patterns of individuals, and, the focus should first be on the effective management of clinical factors, followed by unhealthy lifestyle factors, increased BMI, urban residence, psychological factors, and low/middle SES. Notably, the number of CVD cases that would have been prevented if all factors had been managed, was similar in males and females (PAF: 70%, GIF: 55% in males and PAF: 73%, GIF: 59% in females), but differed significantly by age (i.e., PAF: 58%, GIF: 45% in participants younger than 45 years old and PAF: 87%, GIF: 73% in participants older than 45 years old), implying that for younger participants other understudied factors, such as sleep and peer pressure or support, might better explain the increased burden of CVD (23–25). Other environmental risk factors such as air pollution might also explain this age difference (26).

### 4.2 Findings from other similar studies

When comparing our results with other studies, it should be taken into account that results on PAF and GIF vary between studies, mainly due to the different factors used for the combined PAF calculation, the definition of the outcome, the percentage of exposure removal (for GIF) or population characteristics such as age, sex and race (2, 3). In general, our results are in accordance with other studies conducted (3–5, 22, 26–28).

Investigators from the Global Burden of Disease study have evaluated the burden of 87 risk factors in more than 200 countries/territories during a thirty-year period (i.e., 1990–2019) with varied PAF when combining different factors (4). Of note, between 2010 and 2019, elevated fasting plasma glucose levels, and increased BMI, among others, were associated with the greatest rises in risk exposure, with CVD being the major disease outcome (4). The Global Cardiovascular Risk Consortium, using individual-level data from 1,518,028 individuals (54.1% females, median age 54.4 years) coming from 112 cohort studies, 34 countries, and 8 geographic regions, has found that approximately 57% of incident CVD in females and 53% in males were attributable to 5 modifiable risk factors; smoking, diabetes, BMI, non-high-density lipoprotein cholesterol (non-HDL), systolic blood pressure (SBP) (5). Yusuf et al., studied 155,772 participants of the PURE study from 21 countries and found that approximately 80% of CVD cases for low income, and 70% for middle- and high-income countries, could be attributed to 14 modifiable risk factors (26).

The PURE sub-study from China, found that 12 modifiable risk factors contributed 59% of the PAF for CVD (27). However, contrary to our results, they found higher PAFs for males and rural areas, which could be explained due to racial/genetic differences between European and Asian populations or the fact that the sample comprised of middle-aged adults (age range: 35–70 years) (3). Moreover, an analysis of 226,759 participants of the UK Biobank study, showed that the overall PAF for CVD of 14 modifiable factors declined with age, however, their sample included participants older than 40-years old, which could explain the difference compared to our study (22). Finally, in a recent study by Lee et al, 7 modifiable risk factors (physical inactivity, smoking, poor diet, hypertension, obesity, diabetes, and atrial fibrillation) explained approximately 36% of strokes in females and 33% among males in the US (3); although the percentages were expectedly lower compared to our study (as we studied combined CVD events, stroke included), the results were similar in males and females, similarly to our study. The study investigators also showed lower PAF in younger vs. older participants (25% vs. 50%), further corroborating our results.

### 4.3 Strengths and limitations

The ATTICA study is a long-term prospective cohort study, with 4 assessments, spanning from 2002 (baseline) to 2022, performed by trained health professionals, facilitating a rigorous assessment of the studied modifiable risk factors. Furthermore, the use of the combined PAF and GIF in this study, considers the synergistic or antagonistic relationships between the studied risk factors. Moreover, although the combined PAF has been used before in other studies, the novelty of the current study lies in the idea of focusing on different lifestyle patterns (represented by different combinations of modifiable risk factors), stratifying the analyses by sex and age, the two most prominent non-modifiable risk factors. Finally, we used Miettinen's formula, which uses adjusted relative risks, contrary to Levin's formula, which uses unadjusted relative risks (2).

However, some limitations of the current study should also be acknowledged. Body weight and diet quality were approximated based on the whole 20-year period (2002–2022), hence, in some cases, the outcome (CVD development), preceded these exposures, and, subsequently, a cause-and-effect relationship cannot be inferred. Additionally, PAF simulates the complete eradication of the modifiable risk factor(s); for a more realistic scenario of modifiable risk factor management, we also computed the GIF, set at a percentage of exposure removal of 70% for all studied factors (2, 29). Moreover, even though we studied the most prominent modifiable risk factors, in multi-adjusted models, some residual confounding might still exist.

### 4.4 Conclusion

This study highlights that the increased burden of CVD could be significantly reduced by managing modifiable risk factors and lifestyle patterns, with tailored programs, focusing, firstly, on clinical factors (i.e., diabetes, hypercholesterolemia, hypertension, CKD), secondly, on unhealthy lifestyle factors (i.e., smoking, physical inactivity, low diet quality), and increased BMI, followed by factors such as urban residence, psychological factors, and low/middle SES. Such public health actions should be performed equally in males and females, and interventions should most importantly focus in participants older than 45 years-old, where the burden is larger (and better explained by the studied factors) compared to their younger counterparts. Future research on lifestyle patterns, especially for younger ages, is warranted.

## Data Availability

The datasets generated during and/or analysed during the current study are available from the corresponding author on reasonable request.
